# Early and mid-term outcomes of transcatheter closure of perimembranous ventricular septal defects using double-disc occluders

**DOI:** 10.3389/fcvm.2025.1540595

**Published:** 2025-03-28

**Authors:** Nguyen Cong Ha, Nguyen Lan Viet, Huong Lan Thi Nguyen, Nguyen Sinh Hien, Nguyen Duc Hung

**Affiliations:** ^1^ Hanoi Heart Hospital, Hanoi, Vietnam; ^2^ Department of Cardiology, Hanoi Medical University, Hanoi, Vietnam; ^3^ Institute for Global Health Innovations, Duy Tan University, Da Nang, Vietnam

**Keywords:** ventricular septal defect, transcatheter closure, double-disc occluder, congenital heart disease, procedural outcomes

## Abstract

**Introduction:**

Ventricular septal defect (VSD) is a common congenital heart disease (CHD), accounting for 20–30% of all CHD cases. While surgical closure has been the gold standard for treatment, transcatheter closure has emerged as a less invasive alternative, particularly for perimembranous VSDs. This study aimed to evaluate the early and mid-term outcomes of transcatheter closure using a double-disc occluder device in a single-center Vietnamese cohort.

**Method:**

A prospective descriptive study was conducted at Hanoi Heart Hospital, Vietnam. A total of 81 patients aged ≥1 year or weighing ≥8 kg, with perimembranous VSDs and left-to-right shunting confirmed by Doppler echocardiography, underwent transcatheter closure. Procedural success, complications, and follow-up outcomes were assessed at 1, 3, 12, and 18 months post-procedure. Data were analyzed using SPSS 20.0.

**Results:**

The procedure achieved a success rate of 96.4%, with three failures due to large defects near the aortic valve causing significant aortic regurgitation or residual shunting. No mortality or severe complications such as device embolization or complete atrioventricular block were observed. Minor complications included transient arrhythmias (6.2%), femoral hematoma (8.6%), and mild allergic reactions to contrast agents (9.9%). At 18 months follow-up, residual shunting was minimal in 1.2% of patients, and no cases required surgical intervention.

**Discussion:**

Transcatheter closure of perimembranous VSD using a double-disc occluder device is a feasible, safe, and effective alternative to surgery with high success rates and low complication rates. This approach offers a promising solution for select patients, especially in resource-limited settings. Further multicenter studies are recommended to validate these findings and assess long-term outcomes.

## 1 Introduction

Ventricular septal defect (VSD) is one of the most common congenital heart diseases (1, 2), accounting for approximately 20%–30% of cases (3). Over decades, various surgical and interventional approaches have been developed to manage VSD, including the traditional open-heart surgery with cardiopulmonary bypass, which is considered the gold standard despite associated risks such as infection, neurological complications, and prolonged recovery time.

Since the late 20th century, transcatheter device closure has emerged as a promising alternative for specific types of VSD, including perimembranous VSDs. Early attempts using devices like Rashkind and Button had limitations, such as a high residual shunt rate (25%–60%) and complications affecting adjacent structures like the aortic and tricuspid valves (4). The introduction of the Amplatzer VSD occluder marked a significant advancement; however, concerns over complications such as atrioventricular block (AVB) and device migration limited its widespread use (5, 6).

In recent years, newer devices like the Nit-Occlud® Le VSD-Coils and double-disc occluders have been developed to address these limitations (7). The double-disc occluder, featuring a redesigned structure with smaller and thicker discs, has shown promising results in reducing complications and improving long-term outcomes. International studies have demonstrated high success rates and low complication rates with this device (8–10)^.^

In Vietnam, studies by Nguyen Lan Hieu and others have reported positive outcomes with various devices, including the double-disc occluder, though comprehensive data on its safety and efficacy remain limited (11). These findings highlight the importance of device selection and procedural techniques in achieving optimal outcomes for VSD closure (11, 12). Our study focused on evaluating the short- and mid-term results of perimembranous VSD closure using the double-disc occluder at Hanoi Heart Hospital.

## 2 Materials and methods

### 2.1 Study design and participants

This prospective descriptive study was conducted at Hanoi Heart Hospital to evaluate the early and mid-term outcomes of transcatheter closure of perimembranous VSDs using a double-disc occluder device. Patients eligible for inclusion were those aged ≥1 year or weighing ≥8 kg, diagnosed with perimembranous VSD with a defect diameter >2 mm and left-to-right shunting confirmed by Doppler echocardiography. Inclusion criteria also required patients to have an aortic rim ≥2 mm or aneurysmal membranous septum and at least one of the following: recurrent respiratory infections, failure to thrive, symptomatic heart failure (NYHA classification), left ventricular enlargement (z-score >2.0), or Qp/Qs ratio ≥1.5. Patients were excluded if they had defects >50% of the aortic valve annulus, severe pulmonary hypertension, moderate or severe aortic regurgitation, acquired VSDs, or contraindications such as aspirin intolerance or severe comorbidities. Informed consent was obtained from all participants and their families, and the study adhered to ethical standards approved by the hospital's ethics committee.

### 2.2 Procedure

**Figure 1:**
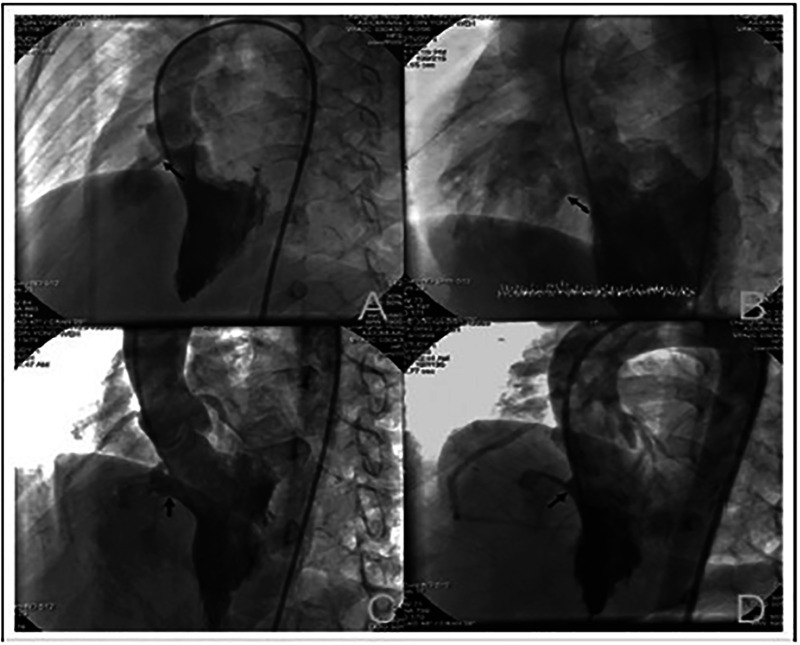
Images of different morphological types of perimembranous ventricular septal defects (VSD) observed on left ventriculography: **(A)** tubular VSD; **(B)** window-like VSD; **(C)** VSD with membranous septal aneurysm; **(D)** funnel-shaped VSD.

The closure procedure was performed under fluoroscopic and transthoracic echocardiographic guidance. General anesthesia or local anesthesia was used based on patient age and cooperation. The femoral artery and vein were accessed using 4–6F sheaths. After initial heparin administration to maintain activated clotting time >200 s, left ventriculography was performed in a left anterior oblique (40–60°) and cranial (20°) projection to assess the VSD's size, morphology, and its relation to the aortic valve. A 0.035-inch hydrophilic guidewire was advanced through the defect to establish an arteriovenous loop. The double-disc occluder was delivered via a catheter using a kissing-wire technique and deployed under fluoroscopic and echocardiographic monitoring to ensure appropriate positioning and exclusion of residual shunting (8–10)^.^ Final assessments included color Doppler imaging and ventriculography to confirm device placement and the absence of significant complications (Figure 1).

### 2.3 Data collection

Baseline demographic and clinical characteristics, including history of recurrent respiratory infections, failure to thrive, and heart failure symptoms, were recorded. Cardiac assessments included electrocardiography, chest x-ray, and transthoracic echocardiography before the procedure. Immediate procedural success was defined by stable clinical status, proper device positioning without displacement, and minimal residual shunting (<2 mm). Patients were followed at 1, 3, 12, and 18 months post-procedure with clinical evaluations, 12-lead electrocardiography, and echocardiography to monitor residual shunting, valvular regurgitation, and other complications.

### 2.4 Statistical analysis

Data were analyzed using SPSS version 20.0. Continuous variables were presented as mean** ±** standard deviation (SD), and categorical variables were expressed as frequencies and percentages. Comparisons between pre- and post-procedure data were conducted using paired *t*-tests for continuous variables and Fisher's exact or chi-square tests for categorical variables. Statistical significance was set at *p* < 0.05.

## 3 Results

The study included 81 patients, with a gender distribution of 53.1% male and 46.9% female. There were 66 patients under 16 years of age, with 49.4% being younger than 6 years and 32.1% aged 6–16 years. Common clinical features included recurrent bronchitis (33.3%) and delayed weight gain (25.9%), particularly prevalent in children under 6 years old. Systolic murmurs were a significant diagnostic feature, with 80.2% having a murmur intensity of ≥3/6. Electrocardiographic findings showed sinus rhythm in all patients, with 2.5% presenting complete right bundle branch block and 34.6% exhibiting left ventricular overload. x-ray imaging revealed pulmonary congestion in 76.5% of patients, and 53.1% had a cardiothoracic index ≥50%. Tricuspid regurgitation was mild in 91.4% of cases, moderate in 3.7%, and severe in 4.9%, while mitral regurgitation was mild in 4.9% of patients, with no regurgitation in the remaining 95.1% (Table 1).

**Table 1:** Demographic and clinical characteristics of patients.

Characteristics		Freq. (*n*)	Percent (%)
Gender	Male	43	53.1
	Female	38	46.9
Age	<6 years	40	49.4
	6–16 years	26	32.1
	>16 years	15	18.5
Recurrent bronchitis		27	33.3
Delayed weight gain		21	25.9
Heart failure symptoms (NYHA II)		11	13.6
Systolic murmur	≥3/6	65	80.2
	<3/6	16	19.8
ECG findings	Sinus rhythm	81	100.0
	Complete right bundle branch block	2	2.5
	Left ventricular overload	28	34.6
X-ray findings	Cardiothoracic index ≥50%	43	53.1
	Pulmonary congestion	62	76.5
Tricuspid regurgitation	Mild (1/4)	74	91.4
	Moderate (2/4)	3	3.7
	Severe (3/4)	4	4.9
Mitral regurgitation	Mild (1/4)	4	4.9
	None	77	95.1

Table 2 shows that after 24 h post-procedure, clinical symptoms significantly improved, with 70.4% of patients having no systolic murmur and 29.6% presenting with a reduced murmur intensity of <3/6. Mild fever (<38.5°C) was observed in 7.4% of patients, while 8.6% had mild hematoma at the puncture site; no severe complications such as arrhythmias, respiratory symptoms, vascular issues, or infections were reported. ECG findings revealed 100% sinus rhythm, with 2.5% of patients showing complete right bundle branch block, which remained unchanged compared to pre-procedural levels, while left ventricular overload decreased from 34.6% pre-procedure to 20.1% post-procedure, indicating improvement. Echocardiographic measures indicated a mean left ventricular end-diastolic diameter of 36.5 mm and a right ventricular diameter of 15.5 mm. Residual shunting was observed in 12.4% of cases, all classified as small, with 87.6% showing complete closure. Mild aortic regurgitation was present in 4.9% of patients, while 97.5% had mild tricuspid regurgitation, with no cases of severe regurgitation. Pulmonary artery systolic pressure averaged 25.3 mmHg. Overall, the procedure demonstrated high safety and effectiveness, with no significant adverse events reported.

**Table 2:** Clinical and diagnostic results after 24 hours post-procedure.

Characteristics		Freq. (*n*)	Percent (%)
Clinical symptoms	Mild fever (<38.5°C)	6	7.4
	No systolic murmur	57	70.4
	Systolic murmur <3/6	24	29.6
	Systolic murmur ≥3/6	0	0
	Arrhythmias	0	0
	Respiratory symptoms (e.g., rales)	0	0
	Allergic symptoms (e.g., rash)	0	0
	Mild hematoma at puncture site	7	8.6
	Limb ischemia or AV fistula at puncture site	0	0
	Oliguria or hematuria	0	0
ECG features	Sinus rhythm	81	100
	Complete right bundle branch block	2	2.5
	Left ventricular overload	17	20.1
	Other arrhythmias	0	0
Echocardiographic measures	LV end-diastolic diameter (mean ± SD, mm)	36.5 ± 8.2	
	RV diameter (mean ± SD, mm)	15.5 ± 3.7	
Doppler findings	Residual shunt (small)	10	12.4
	No residual shunt	71	87.6
	Aortic regurgitation (mild 1/4)	4	4.9
	No aortic regurgitation	77	95.1
	Tricuspid regurgitation (mild 1/4)	79	97.5
	Tricuspid regurgitation (moderate 2/4–3/4)	2	2.5
	Severe tricuspid regurgitation (4/4)	0	0
Pulmonary artery pressure	Pulmonary artery systolic pressure (mean ± SD, mmHg)	25.3 ± 3.5	
Complications	Mild hematoma at puncture site	7	8.6
	Mild fever resolved in 48 h	5	6.2
	Skin rash	8	9.9
	Other complications	0	0

Over the follow-up periods of 1, 3, 12, and 18 months, clinical and echocardiographic results demonstrated consistent improvement and stability. No patients experienced shock, syncope, or heart failure symptoms (NYHA > I) at any time point. Residual shunting decreased progressively, with 3.7% of patients showing residual shunts at 1 month, reducing to 2.5% at 3 months and 1.2% at 12 and 18 months. Mild aortic regurgitation persisted in 4.9% of patients throughout, while mild tricuspid regurgitation was observed in 97.5%, with moderate-to-severe cases limited to 2.5%. Left ventricular overload decreased from 9.9% at 1 month to 3.7% at 3 months and remained stable thereafter. Pulmonary artery systolic pressure showed a slight decrease over time, averaging 24.3 mmHg at 1 month and 23.5 mmHg by 12 and 18 months. Similarly, the left ventricular end-diastolic diameter remained stable, averaging 34.4 mm at 1 month and 35.1 mm at 12 and 18 months. These findings indicate sustained clinical and hemodynamic improvements with no significant adverse events or progression of regurgitation over the follow-up periods (Table 3).

**Table 3:** Comparison of clinical and echocardiographic results over time.

Variables	1 month (*n*, %)	3 months (*n*, %)	12 months (*n*, %)	18 months (*n*, %)
Clinical symptoms (shock, syncope)	0 (0%)	0 (0%)	0 (0%)	0 (0%)
Heart failure symptoms (NYHA > I)	0 (0%)	0 (0%)	0 (0%)	0 (0%)
Systolic murmur ≥3/6	0 (0%)	0 (0%)	0 (0%)	0 (0%)
Residual shunt	3 (3.7%)	2 (2.5%)	1 (1.2%)	1 (1.2%)
Mild aortic regurgitation (HoC)	4 (4.9%)	4 (4.9%)	4 (4.9%)	4 (4.9%)
Mild tricuspid regurgitation (HoBL)	79 (97.5%)	79 (97.5%)	79 (97.5%)	79 (97.5%)
Moderate-to-severe tricuspid regurgitation	2 (2.5%)	2 (2.5%)	2 (2.5%)	2 (2.5%)
Left ventricular overload	8 (9.9%)	3 (3.7%)	3 (3.7%)	3 (3.7%)
Pulmonary artery systolic pressure (mmHg)	24.3 ± 3.3	25.5 ± 3.4	23.5 ± 3.4	23.5 ± 3.4
Left ventricular end-diastolic diameter (Dd, mm)	34.4 ± 7.2	33.4 ± 6.7	35.1 ± 7.3	35.1 ± 7.3

Table 4 summarizes the association between residual shunt presence and various factors, including defect morphology, defect size, and device size. Residual shunts were most common in window-like morphology (100%), followed by funnel-shaped defects with aneurysm (81.2%) and tubular morphology (55.5%). However, there was no statistically significant difference in residual shunts across morphologies (*p* = 0.259). Residual shunt prevalence increased with larger defect sizes, observed in 62.5% of defects sized 3–4 mm and reaching 100% in defects sized 5–8 mm, with a statistically significant difference (*p* = 0.040). Regarding device size, residual shunts were less frequent in smaller devices, seen in 50% of cases with size 4 devices, increasing to 73.1% with size 5 devices and 100% in devices sized 7, 8, and 10, showing a significant association (*p* = 0.028). These results indicate that residual shunts are influenced by defect size and device size but not significantly by defect morphology.

**Table 4:** Residual shunt according to several factors.

Group	Category	Residual shunt (*n*, %)	No residual shunt (*n*, %)	*p*-value
Morphology	Tubular	5 (55.5%)	4 (44.5%)	0.259
	Window-like	3 (100%)	0 (0%)	
	Funnel-shaped with aneurysm	56 (81.2%)	13 (18.8%)	
Defect size (mm)	3–4	20 (62.5%)	12 (37.5%)	0.040
	4–5	29 (85.3%)	5 (14.7%)	
	5–6	11 (100%)	0 (0%)	
	6–7	3 (100%)	0 (0%)	
	7–8	1 (100%)	0 (0%)	
Device size	4	8 (50%)	8 (50%)	0.028
	5	19 (73.1%)	6 (26.9%)	
	6	19 (86.4%)	3 (13.6%)	
	7	11 (100%)	0 (0%)	
	8	6 (100%)	0 (0%)	
	10	1 (100%)	0 (0%)	

Table 5 summarizes the outcomes, challenges, and complications associated with the procedure. The success rate was 96.4%, with only 3.6% of cases failing due to large ventricular septal defect (VSD) sizes and proximity to the aortic valve causing significant residual shunts and aortic regurgitation. Mild complications included respiratory symptoms (6.2%), resolved with manual ventilation, and mild fever (7.4%), resolved within 48 h without infection. Contrast agent allergies occurred in 9.9% of patients, presenting as skin rashes that resolved within 24 h. Procedural challenges included difficulty crossing the VSD (24.7%), snare re-captures (22.2%), and switching to larger devices (16%) due to significant shunting risks. Device delivery issues led to restarting the procedure in 11.1% of cases. Transient ventricular tachycardia was observed in 21.8% of cases during wire manipulation but resolved without hemodynamic impact. Temporary first- or second-degree atrioventricular block (2.5%) and sinus bradycardia (3.7%) were also noted. One patient (1.2%) experienced worsened tricuspid regurgitation, and another had a small residual shunt (1–2 mm) after 18 months. Overall, complications were manageable, with no severe or life-threatening events reported.

**Table 5:** Summary of success rates, failures, challenges, and complications.

Characteristics	*n*	%	Causes/remarks
Success rate	81/84	96.4%	
Failure rate	3/84	3.6%	Due to large VSD size, proximity to the aortic valve causing aortic regurgitation and large residual shunts.
Respiratory symptoms (mild)	5	6.2%	Required manual ventilation support, no intubation necessary.
Vascular access complications	7	8.6%	Mild hematoma at access site; no major bleeding or vascular occlusion observed.
Mild fever (<38.5°C)	6	7.4%	Resolved within 48 h; no infections in respiratory tract, access site, or endocarditis.
Contrast agent allergy (Hexabrix 320)	8	9.9%	Skin rash; no anaphylaxis, resolved within 24 h.
Difficulty crossing VSD	20	24.7%	Initial use of IM catheter or Terumo hydrophilic guidewire, later switched to JR or pigtail catheter.
Snare re-capture	18	22.2%	Obstruction by chordae tendineae or difficulty in passing delivery system through the VSD.
Switching to larger device	13	16.0%	Due to large shunts, increasing risk of residual shunt and complications.
Restarting procedure	9	11.1%	Device slipped into the right ventricle during delivery system retrieval through the aortic valve.
Transient ventricular tachycardia	17	21.8%	Occurred during catheter or wire manipulation; resolved immediately without hemodynamic impact.
Temporary 1st/2nd Degree AV block	2	2.5%	
Sinus bradycardia	3	3.7%	
Worsened tricuspid regurgitation	1	1.2%	Increased from 1/4 to 2–3/4 severity.
Residual shunt after 18 months	1	1.2%	Small shunt (1–2 mm); patient had a 4 mm defect, window-like morphology, and a size 6 device.

## 4 Discussion

The study demonstrated a high procedural success rate with minimal complications and manageable challenges. Residual shunting was rare and primarily linked to larger defect sizes, specific morphologies, or device mismatches. Transient complications such as mild respiratory symptoms, fever, and contrast agent allergies resolved without significant intervention. Procedural challenges, including difficulty crossing the VSD, device snare re-captures, and the need for larger devices, were effectively managed. Serious complications, such as transient arrhythmias, were infrequent and self-limiting. Long-term outcomes showed stable clinical and echocardiographic improvements, emphasizing the efficacy and safety of transcatheter VSD closure.

The study highlights key procedural characteristics and outcomes of transcatheter closure for VSDs. A left oblique view of 40–60° with a cranial tilt of 20° was universally used for imaging during the procedure, aligning with practices in the EUREVECO study by Haas et al., which also employed similar imaging angles and snaring techniques (13). Crossing the VSD was most successful using a 0.035 hydrophilic guidewire (58%), while the inner mammary catheter (28%), pigtail catheter (11%), and JR catheter (3%) were less frequently used. The kissing technique was applied in all cases for delivering devices, with the snare position predominantly in the superior or inferior vena cava (66.7%) and device deployment initiated from the left ventricle in 75.3% of cases. Residual shunting was observed in 79% of cases immediately post-deployment, higher than rates reported by Lei Wang (45.1%) and Haas (56.9%) (13), though similar to the findings of Nguyen Lan Hieu, depending on device type (11). Evaluation via left ventriculography and transthoracic echocardiography was essential in assessing device position and residual shunting.

Surgical closure remains the standard treatment for VSDs but involves inherent risks such as the use of cardiopulmonary bypass and atrioventricular block (1%–2%) (3, 4, 14, 15). Transcatheter interventions offer advantages including reduced hospital stay, lower costs, minimal myocardial damage, and a comparable efficacy in selected cases (14, 15). However, device-specific challenges persist. Amplatzer devices show high rates of atrioventricular block (BAV), while Coils are better suited for small defects but present higher rates of hemolysis and residual shunts. Ductal occluders are prone to displacement, whereas double-disc devices demonstrate fewer complications such as device dislodgment, residual shunting, and hemolysis. Although no device completely eliminates the risk of BAV, the complication rates are comparable to those of surgical repair, underscoring the importance of device selection and procedural expertise.

The success rate of transcatheter closure of ventricular septal defects (VSDs) in this study was 96.4%, aligning with findings from other researchers, such as Nguyen Lan Hieu (DO: 95.6%; Coils: 97.2%) (11), and Linqi Yang et al., whose meta-analysis of 4,406 cases reported a pooled success rate of 96.6% (range 83.3%–100%) (16). The primary causes of failure were related to anatomical factors, including large defects near the aortic valve causing regurgitation (1.2% in this study), large residual shunts, and challenges in advancing the device through the defect. Comparative data indicate that device deployment failures occurred in 1.5% of cases in Lei Wang's study (17) and 0.9% in EUREVECO (13), whereas none were observed in this study. Similarly, complications like significant residual shunting (1.2%) and severe aortic regurgitation (1.2%) were on par with or better than rates reported by other studies, such as EUREVECO (1.8%) (13) and Nguyen Lan Hieu (DO: 0.3%, Coils: 1.4%) (11).

Complications leading to termination of procedures included severe arrhythmias, particularly atrioventricular block (BAV), with rates varying across studies: 0.9% in EUREVECO [1], 0.3% in Nguyen Lan Hieu's DO group (11), and none in Lei Wang's study (17) or this study. Other procedural challenges, such as tricuspid regurgitation or anatomical incompatibilities, were infrequent across studies. For instance, EUREVECO reported 1.8% of cases where Coils could not be positioned correctly (13), while Nguyen Lan Hieu observed a 1.4% failure rate in the Coils group due to anatomical incompatibilities (11). This study's findings confirm the feasibility and safety of transcatheter VSD closure while emphasizing the importance of careful patient selection and procedural planning to address anatomical and technical challenges.

The study revealed no severe complications like mortality, device embolization, or significant hemolysis, consistent with findings by Nguyen Lan Hieu (11) or Lei Wang (17). However, residual complications, such as severe tricuspid regurgitation (1.2%) and minor device-related challenges, were noted. Across studies, severe arrhythmias, particularly third-degree atrioventricular block (BAV), were infrequent but significant. In this study, no cases of BAV were reported, whereas Lei Wang documented one case (0.2%) requiring permanent pacing (17), and Haas reported a recoverable case with corticosteroids (13). Similarly, device embolization necessitating surgical retrieval occurred in 0.7% of cases in Linqi Yang's meta-analysis (16), but not in this study. These findings highlight the overall safety of the transcatheter VSD closure, with rare but manageable severe complications.

Mild complications ranged between 10%–30% across studies, including hematomas at the access site, minor hemolysis, and transient arrhythmias. This study reported a higher incidence (38.3%) of mild complications, including access-site hematomas (8.6%) and transient allergic reactions to contrast agents (9.9%), which were self-limiting. Comparatively, Nguyen Lan Hieu observed 12.1% in the DO group and 14.5% in the Coils group, with hemolysis more prevalent in Coils (7.3%) (11). Haas reported a 14.7% rate of mild complications, primarily right bundle branch block (5.9%) and mild tricuspid regurgitation (4.9%) (13). Lei Wang recorded a 19.8% rate, including 0.19% device misplacement requiring snare retrieval (17). The findings emphasize the need for meticulous procedural planning to minimize such events.

From the study results and years of procedural experience, several key recommendations were identified to enhance the success and minimize complications of perimembranous ventricular septal defect (VSD) closure using a double-disc device. Patient selection is critical: candidates should be over one year old, with defect sizes ranging from 3 to 8 mm, an aortic rim ≥1–2 mm or aneurysmal membranous septum, moderate left-to-right shunt, and normal or mildly elevated pulmonary artery pressure. The femoral artery and vein are the most suitable access routes, with either local anesthesia or intravenous sedation being effective. Optimal imaging angles (40–60° left anterior oblique and 20° cranial) are essential for evaluating VSD morphology, and final closure decisions should be based on angiographic findings, favoring defects with an aortic rim ≥2 mm or aneurysmal features. Using a 0.035-inch hydrophilic curved wire is advantageous for crossing the defect, minimizing arrhythmia risk, and ensuring high success rates; catheters may be used if this approach fails. Snaring in the superior vena cava is preferred over the pulmonary artery for creating arteriovenous loops. Device release from the left ventricle offers stability and reduces system displacement into the right ventricle, especially for less experienced operators. Device sizing should exceed the smallest angiographic defect diameter by 2 mm, and in cases of aneurysmal septa or window-like defects, up to 3 mm. Procedural support from transthoracic echocardiography and a 3–5 day hospital observation post-procedure are advised to monitor for atrioventricular block, which commonly occurs within the first week.

Despite the promising findings and recommendations, this study has several limitations. The relatively small sample size (*n* = 81) may limit the generalizability of the results to broader populations. The study was conducted in a single-center setting, which may introduce biases related to specific procedural techniques or operator experience. Additionally, the follow-up period, while sufficient to observe early complications, may not capture long-term outcomes such as late device migration, progressive valve regurgitation, or delayed onset of arrhythmias. The study primarily relied on transthoracic echocardiography to guide the procedure and assess aortic regurgitation, tricuspid regurgitation, and residual shunting. However, the absence of a centralized echocardiographic core lab may have affected the consistency of valvular dysfunction and shunt assessments. Furthermore, while transthoracic echocardiography and angiography are effective, they may not fully evaluate subtle morphological variations or long-term hemodynamic changes. Finally, the lack of a control group undergoing surgical repair or alternative closure methods limits the ability to compare efficacy and safety outcomes across different approaches. Future multicenter studies with larger sample sizes, longer follow-up periods, and standardized imaging assessments are needed to validate these findings and address these limitations.

## 5 Conclusion

In conclusion, the study demonstrates that transcatheter closure of perimembranous ventricular septal defects (VSD) using a double-disc device is a highly effective and safe alternative to surgical repair for appropriately selected patients. With a success rate of 96.4% and a low incidence of severe complications, this method offers significant advantages, including reduced recovery time and fewer procedural risks. Key factors contributing to successful outcomes include careful patient selection, optimized procedural techniques, appropriate device sizing, and effective imaging and guidance during the procedure. While the findings highlight the potential of this approach, limitations such as a small sample size, single-center design, and short follow-up period underscore the need for further multicenter, longitudinal studies to validate these results and explore long-term outcomes. These findings contribute to the growing evidence supporting transcatheter closure as a viable and less invasive option for managing perimembranous VSDs.

##  Data availability statement

The raw data supporting the conclusions of this article will be made available by the authors, without undue reservation.

## Ethics statement

The studies involving humans were approved by Institutional review board of Hanoi Heart Hospital. The studies were conducted in accordance with the local legislation and institutional requirements. Written informed consent for participation in this study was provided by the participants’ legal guardians/next of kin.
